# Wheatgerm agglutinin-mediated toxicity in pancreatic cancer cells

**DOI:** 10.1038/sj.bjc.6690593

**Published:** 1999-08

**Authors:** R E Schwarz, D C Wojciechowicz, A I Picon, M A Schwarz, P B Paty

**Affiliations:** Department of Surgery, Memorial Sloan-Kettering Cancer Center, New York, NY 10021, USA; Department of Pediatrics, Columbia-Presbyterian Medical Center,630 West 168th Street,New York, NY 10032, USA

**Keywords:** wheatgerm agglutinin, pancreatic cancer, lectin, apoptosis, sialic acid surface binding, swainsonine

## Abstract

Lectin binding specificities for carbohydrate allow phenotypic and functional characterization of membrane-associated glycoproteins expressed on cancer cells. This analysis examined wheatgerm agglutinin binding to pancreatic cancer cells in vitro and the resulting toxicity. Membrane preparations of nine human pancreatic carcinoma cell lines were studied for lectin binding using wheatgerm agglutinin (WGA), concanavalin A (ConA) and phytohaemagglutinin-L (PHA-L) in a lectin blot analysis. Cell proliferation in vitro was measured by thymidine incorporation in the absence or presence of lectins at various concentrations. Sialic acid binding lectins or succinyl-WGA (succWGA) served as controls. WGA toxicity was tested after swainsonine or neuraminidase pretreatment. Binding and uptake of fluorescein-labelled lectins was studied under fluorescence microscopy. All pancreatic cell lines displayed high WGA membrane binding, primarily to sialic acid residues. Other lectins were bound with weak to moderate intensity only. Lectin toxicity corresponded to membrane binding intensity, and was profound in case of WGA (ID_50_ at 2.5–5 μg ml^−1^). WGA exposure induced chromatin condensation, nuclear fragmentation and DNA release consistent with apoptosis. Important steps for WGA toxicity included binding to sialic acid on swainsonine-sensitive carbohydrate and lectin internalization. There was rapid cellular uptake and subsequent nuclear relocalization of WGA. In contradistinction to the other lectins studied, WGA proved highly toxic to human pancreatic carcinoma cells in vitro. WGA binding to sialic acid residues of N-linked carbohydrate, cellular uptake and subsequent affinity to N-acetyl glucosamine appear to be necessary steps. Further analysis of this mechanism of profound toxicity may provide insight relevant to the treatment of pancreatic cancer. © 1999 Cancer Research Campaign


*British Journal of Cancer* (1999) **80**(11), 1754–1762
© 1999 Cancer Research Campaign
Article no. bjoc.1998.0593

### Wheatgerm agglutinin-mediated toxicity in pancreatic cancer cells

**RE Schwarz[1], DC Wojciechowicz[1], AI Picon[1], MA Schwarz[2]* and PB Paty[1]**

[1]Department of Surgery, Memorial Sloan-Kettering Cancer Center, 1275 York Avenue, New York, NY 10021, USA; [2]Department of Pediatrics, Columbia-Presbyterian Medical Center, 630 West 168th Street, New York, NY 10032, USA

**Summary** Lectin binding specificities for carbohydrate allow phenotypic and functional characterization of membrane-associated glycoproteins expressed on cancer cells. This analysis examined wheatgerm agglutinin binding to pancreatic cancer cells in vitro and the resulting toxicity. Membrane preparations of nine human pancreatic carcinoma cell lines were studied for lectin binding using wheatgerm agglutinin (WGA), concanavalin A (ConA) and phytohaemagglutinin-L (PHA-L) in a lectin blot analysis. Cell proliferation in vitro was measured by thymidine incorporation in the absence or presence of lectins at various concentrations. Sialic acid binding lectins or succinyl-WGA (succWGA) served as controls. WGA toxicity was tested after swainsonine or neuraminidase pretreatment. Binding and uptake of fluorescein-labelled lectins was studied under fluorescence microscopy. All pancreatic cell lines displayed high WGA membrane binding, primarily to sialic acid residues. Other lectins were bound with weak to moderate intensity only. Lectin toxicity corresponded to membrane binding intensity, and was profound in case of WGA ($ID_{50}$ at 2.5–5 µg $ml^{-1}$). WGA exposure induced chromatin condensation, nuclear fragmentation and DNA release consistent with apoptosis. Important steps for WGA toxicity included binding to sialic acid on swainsonine-sensitive carbohydrate and lectin internalization. There was rapid cellular uptake and subsequent nuclear relocalization of WGA. In contradistinction to the other lectins studied, WGA proved highly toxic to human pancreatic carcinoma cells in vitro. WGA binding to sialic acid residues of N-linked carbohydrate, cellular uptake and subsequent affinity to N-acetyl glucosamine appear to be necessary steps. Further analysis of this mechanism of profound toxicity may provide insight relevant to the treatment of pancreatic cancer.

**Keywords:** wheatgerm agglutinin; pancreatic cancer; lectin; apoptosis; sialic acid surface binding; swainsonine

Alterations in cell surface carbohydrate on cancer cells have been linked to increased metastatic capabilities and more aggressive biologic behaviour (Dennis and Laferte, 1987; Dennis et al, 1987; Gorelik et al, 1995). We have previously shown a correlation of cancer-specific, N-linked β1-6 branched carbohydrate and K-*ras* protein activation in both colorectal and pancreatic cancer cells (Wojciechowicz et al, 1995; Schwarz et al, 1996). In either system, cell surface expression of N-linked β1-6 branched carbohydrate correlated directly with in vitro susceptibility to phytohaemagglutinin-L (PHA-L) toxicity, a lectin with specific binding properties to β1-6 branched N-linked carbohydrate. Since pancreatic carcinoma cells express abundant sialic acid residues on cell surface carbohydrate (Maylie-Pfenninger and Jamieson, 1979; Sowa et al, 1987; Ching et al, 1988; Ho et al, 1988; Willemer et al, 1990), we were interested in the functional binding characteristics of wheatgerm agglutinin (WGA), a lectin with specific binding properties to sialic acid and N-acetyl glucosamine (GlcNAc) (Monsigny et al, 1980; Wright, 1992). This study shows profound in vitro toxicity of WGA in all pancreatic cancer lines tested. The effect appears to be carbohydrate-mediated, limited by initial sialic acid binding as well as lectin internalization, but also dependent on GlcNAc binding ability.

*Received 13 November 1997*
*Revised 4 August 1998*
*Accepted 5 August 1998*

*Correspondence to:* RE Schwarz, Department of General Oncologic Surgery, City of Hope National Medical Center, 1500 East Duarte Road, Duarte, CA 91010-3000, USA

### MATERIALS AND METHODS

### Cell culture

Nine human pancreatic carcinoma cell lines obtained from the American Tissue Culture Collection (ATCC) were maintained in vitro under standard tissue culture conditions. They included BxPC, MIA, Panc-1, CFPAC, ASPC, HS-766T, HTB-147, CaPan-1 and CaPan-2. All lines were grown at 37°C and 5% carbon dioxide in sterile RPMI-1640 medium with 10% fetal bovine serum after addition of glutamate, penicillin (50 U $ml^{-1}$) and streptomycin (50 µg $ml^{-1}$) (complete medium). Cells were grown on standard tissue culture plastic flasks to 80% confluency and passed after trypsinization.

### Lectins

Tissue culture-grade or conjugated lectins were dissolved in sterile phosphate-buffered saline (PBS) without additives and kept at 4°C in a concentration of 1 mg $ml^{-1}$. Wheatgerm agglutinin (WGA, specific for sialic acid and GlcNAc), Concanavalin A (ConA, specific for α-D-mannose and α-D-glucose), and phytohaemagglutinin L (PHA-L, specific for the β1-6 branch at the trimannosyl core of N-linked carbohydrate) were obtained from Sigma Chemicals. Succinyl-WGA (succWGA, specific only for GlcNAc but not for sialic acid (Monsigny *et al*, 1979)) was purchased from

*Present address: Department of Pediatrics, Los Angeles Childrens Hospital, 4650 Sunset Boulevard, Los Angeles, CA 90027, USA

Vector Laboratories. Control lectins for sialic acid binding were *Maackia amurensis* agglutinin (MAA), *Sambucus nigra* agglutinin (SNA) and *Limulus polyphemus* agglutinin (LPA), all purchased from Sigma Chemicals.

### Lectin blots

For membrane isolation, cells were scraped from the tissue culture dish, washed in cold PBS, and snap frozen at –70°C. Membrane fractions for lectin blot analysis were prepared by dounce homogenization as described earlier (Wojciechowicz *et al*, 1995; Schwarz *et al*, 1996). Briefly, after two centrifugation steps at 600***g*** for 5 min and at 16 000***g*** for 10 min, the resulting pellet of total cellular membrane was solubilized in 200 µl of PBS containing 1% NP-40, 2 mM EDTA, 1 mM phenylmethyl sulphoxide (PMSF), 100 µg ml$^{-1}$ aprotinin and leupeptin and 50 µg ml$^{-1}$ pepstatin. Neuraminidase treatment of some membrane samples was performed with 2 mU of *Vibrio cholerae* neuraminidase (Boehringer Mannheim) per 2.5 µg of solubilized membrane protein at 37°C overnight. Membrane proteins (10 µg) were separated by sodium dodecyl sulphate polyacrylamide gel electrophoresis (SDS-PAGE) under reducing conditions (Laemmli, 1970), transferred to nitrocellulose (Towbin *et al*, 1979), and the membrane blocked for 4 h in 0.1% Tween-20 in Tris-buffered saline containing 0.1 mM of calcium chloride and manganese chloride (buffer A) with addition of 3% (w/v) bovine serum albumin. For lectin binding, the membrane was incubated for 1 h in buffer A with 1 µg ml$^{-1}$ of lectin conjugated with horseradish peroxidase (HRP). Bound lectin was identified with a chemilluminescent detection system (ECL, Amersham Life Science Inc.). For reprobing, the membranes were stripped over night with buffer A containing 0.1% (w/v) SDS.

### Cell proliferation

After trypsinization, cells were washed twice in complete medium, resuspended and counted in a haemocytometer. After placement into a 96-well flat-bottom tissue culture plate at a density of 0.5–1.5 × $10^4$ cells per well, the cells were allowed to equilibrate and readhere over 6 h at 37°C. Lectins were then added in complete medium at final concentrations of 1–500 µg ml$^{-1}$. All experimental measurements were performed in triplicates or quadruplicates. After incubation over 48 h, $^3$H-labelled thymidine was added at 0.5 µCi per well, and the cells harvested into glass fibre filter membranes (Titertek) after another 6 h of incubation. Subsequent to addition of scintillation fluid, radioactive uptake of the filter membranes was measured in a β-counter with automatic quench adjustment (Beckman). Group data were calculated as mean counts per minute, and expressed as proliferation relative to control (in per cent). All group standard deviations fell within ± 4% of the mean.

### Chromatin and DNA labelling analysis

Cells were plated at 5 × $10^4$ cells per well on 8-chamber plastic tissue culture microscopy plates (Nunc) and incubated overnight in complete medium. Lectins were added at 20 µg ml$^{-1}$ as described for a total incubation of 48 h. The plates were washed with PBS and fixed in 2% paraformaldehyde (w/v) for 10 min. After additional washes, 6-diamidino-2-phenylindole-dilactate (DAPI) was added at 2.5 µg ml$^{-1}$ for 10 min for chromatin labelling (Molecular Probes). The slides were rinsed with PBS, mounted with 10% glycerol in PBS (v/v), sealed with a cover slip, and examined under a fluorescence microscope (Olympus). For a cellular DNA fragmentation enzyme-linked immunosorbent assay (ELISA) analysis, a commercial 5′-Bromo-2′-deoxyuridine (BrdU) labelling kit was used (Boehringer Mannheim). Cells were labelled with BrdU overnight, plated in microtitre well plates at 1 × $10^4$ cells per well, and exposed to lectins for up to 12 h. After centrifugation at 250***g*** for 10 min, supernatant was transferred to a microtitre plate precoated with anti-DNA antibody (Boehringer Mannheim). After three cycles of washing and microwave denaturing, 100 µl of anti-BrdU–peroxidase solution were added, incubated for 90 min at room temperature, and 100 µl of TMB substrate solution were added. The reaction was stopped with 25 µl of $H_2SO_4$ solution (16% v/v), and a photometric analysis performed at 450 nm.

### Cell pretreatment before proliferation analysis

For some WGA proliferation experiments, cells were pretreated with swainsonine or neuraminidase along modifications of standard protocols (Maylie-Pfenninger and Jamieson, 1979; Dennis, 1986*b*). Swainsonine, an α-mannosidase-2 inhibitor that causes sialyllactosamine inhibition on N-linked carbohydrate and leads to formation of hybrid carbohydrate truncations, was added to the tissue culture plates at 0.03 or 0.3 µg ml$^{-1}$ over 12 h (Dennis, 1989*b*; Dennis *et al*, 1989; Yagel *et al*, 1989). Cells were then washed, and lectins added as described. In cases of neuraminidase pretreatment, suspended cells were incubated at 2 × $10^6$ per ml in serum-free medium with neuraminidase from *Vibrio cholerae* at 50 mU ml$^{-1}$ and 37°C over 1 h. The cells were then washed twice with complete medium, checked for viability with trypan blue, counted and plated into 96-well plates as described. For experiments involving immobilized lectin, a technique modified after McClay et al (1981) was used. WGA in PBS solution at 2–80 µg ml$^{-1}$ was loaded into uncoated 96-well plastic plates at 50 µl per well for 1 h at room temperature. The plates were flicked, washed four times with PBS, loaded with 3% bovine serum albumin in PBS (w/v) for 1 h at room temperature at 250 µl per well, and washed again with PBS. Cells were then added in complete medium for proliferation analysis as described.

### Fluorescent microscopy with FITC–lectin conjugates

Cells were plated at 5 × $10^4$ cells per well on 8-chamber plastic tissue culture slides (Nunc) and incubated over night in complete medium. The trays were then cooled to 4°C, and fluorescein isothiocyanate (FITC) labelled lectins added to final concentrations of 10 µg ml$^{-1}$. After various incubation intervals at 37°C, the trays were washed twice with PBS, fixed in 2% paraformaldehyde solution in PBS (w/v), rinsed twice, quenched with 50 mM ammonium chloride ($NH_4Cl$) in PBS for 10 min and rinsed again. Cells were permeabilized with a solution consisting of 0.075% saponin in PBS (v/v) and 0.2% bovine serum albumin (w/v) for 30 min at room temperature and rinsed in PBS. Treatment with 5 µg ml$^{-1}$ ribonuclease A (DNAase free) in PBS for 30 min, rinsing and incubation in 5 µg ml$^{-1}$ propidium iodine in PBS for 20 min followed. After rinsing with PBS four times, the slides were mounted with Vectashield (Vector) and the cover slip was sealed with nail polish. Slides were kept in the dark at –20°C. Confocal fluorescent microscopy was performed at the confocal microscopy core facility at Cornell Medical College, New York, NY.

© 1999 Cancer Research Campaign

### RESULTS

### Lectin blots

Lectin blots of crude membrane preparations of nine human pancreatic carcinoma cell lines revealed low binding intensity for PHA-L and low to moderate binding intensity for ConA. We had previously shown that, in the case of PHA-L, surface membrane binding intensity correlated with the degree of K-*ras* activation (Schwarz *et al*, 1996). In contradistinction, WGA membrane binding was strong in all cell lines tested before neuraminidase treatment (Figure 1). After cleavage of sialic acid by neuraminidase pretreatment of the membrane preparations, the WGA binding intensity decreased significantly in all cell lines, with residual WGA binding being variable between cell lines, indicating a strong sialic acid content of membrane glycoproteins in all cell lines, but a variable N-acetylglucosamine content. WGA-reactive glycoproteins were detected in multiple bands between 45 and 200 kDa molecular size on SDS-PAGE analysis.

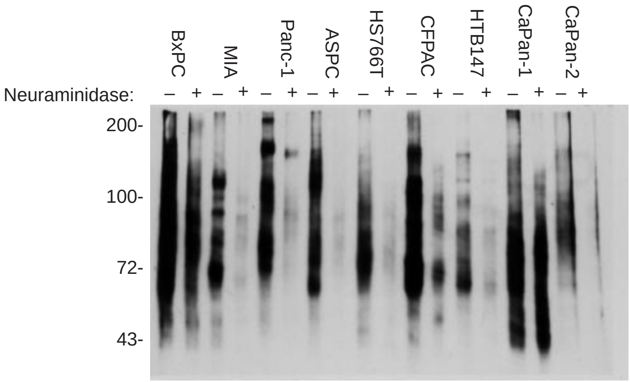

**Figure 1** Wheatgerm agglutinin blot. Membrane isolates of nine human pancreatic cancer cell lines were probed for WGA binding after SDS-PAGE separation. The two columns for each cell line represent untreated (–) versus neuraminidase-pretreated (+) membrane isolates in order to demonstrate WGA binding before or after cleavage of sialic acid

### Cell proliferation and in vitro lectin toxicity

In vitro cell proliferation of all cell lines analysed was affected by addition of lectins in a dose-dependent manner. While cell proliferation was only weakly inhibited by addition of PHA-L, and moderately by addition of ConA, WGA displayed strong toxicity in all nine pancreatic cell lines. For the sake of clarity, only one representative cell line is depicted (Figure 2). A 50% inhibition of proliferation ($IC_{50}$) was observed at WGA concentrations between 2.5 and 7.5 μg $ml^{-1}$, depending on the cell line tested. As reported earlier, this $IC_{50}$ ranged from 7.5 to 28 μg $ml^{-1}$ for ConA, and was not reached for the majority of cell lines at a concentration of 40 μg $ml^{-1}$ in case of PHA-L (Schwarz *et al*, 1996). Thus, it appeared that general susceptibility to in vitro lectin toxicity correlated with the intensity of membrane surface reactivity on lectin blots in all cell lines tested. Both ConA and PHA-L, but not WGA were noted to induce a slight proliferation-stimulating effect at low concentrations of 1.25–2.5 μg $ml^{-1}$. Succinyl-WGA proved to have no effect whatsoever in a dose range up to 40 μg $ml^{-1}$, indicating the importance of WGA binding to sialic acid to induce toxicity at this dose range. When added to the cell proliferation assay at significantly higher concentrations, however, succWGA was able to cause a mild inhibition of proliferation in three cell lines tested, supporting the possibility of cytotoxicity after binding to the relatively sparse GlcNAc moieties of membrane glycoproteins (Figure 3).

### Swainsonine pretreatment

In an attempt to identify the nature of structures that mediate or facilitate WGA toxicity in pancreatic cancer cell lines in vitro, cells were pretreated with swainsonine, an α-mannosidase-2 inhibitor that blocks the addition of polylactosamine antennae and sialic acid to the mannosyl core of N-linked carbohydrate (Dennis, 1986*b*). As shown in Figure 4, pretreatment with swainsonine caused little direct inhibition of proliferation. When swainsonine-pretreated cells were exposed to WGA, however, there was a dose-dependent abrogation of WGA-induced proliferation blockade, indicating that the WGA effect is predominantly mediated through a swainsonine-sensitive structure, e.g. a carbohydrate molecule susceptible to sialyllactosamine inhibition.

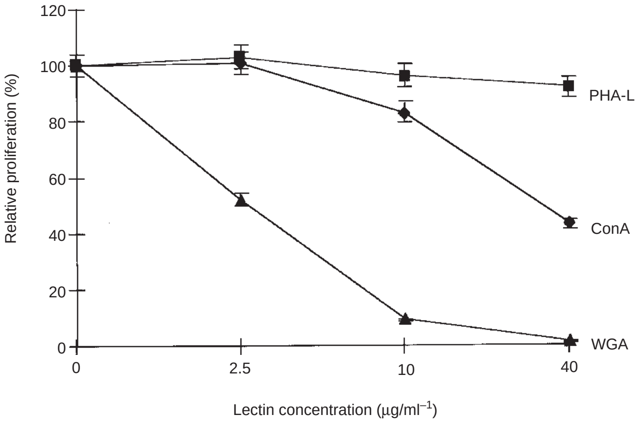

**Figure 2** Lectin toxicity. Proliferation of cell line Panc-1 was measured by thymidine incorporation after a 48-h exposure to lectins (Concanavalin A, phytohaemagglutinin-L, wheatgerm agglutinin) at various concentrations. Bars depict standard deviation

### Neuraminidase pretreatment

Lectin toxicity after target cell pretreatment with neuraminidase was investigated in three cell lines (BxPC, MIA, Panc-1). After a 1-h exposure to neuraminidase at 37°C, the viability was greater than 97% in all cell lines. Comparison of proliferation by neuraminidase-treated versus untreated cells revealed no significant difference at 48 h. After addition of WGA, however, neuraminidase-pretreated cells showed a much lesser degree of toxicity than untreated controls (Figure 5). Neuraminidase pretreatment did not lead to a complete abrogation of WGA toxicity at WGA doses of 5 and 20 μg $ml^{-1}$. When other lectins were studied after neuraminidase cell treatment, a slight increase in toxicity was observed in pretreated cells after addition of ConA, PHA-L, and succWGA (data not shown).

### DAPI and BrdU analysis

DAPI stains of pancreatic cancer cells after in vitro exposure to WGA revealed the presence of multiple nucleosomes with chromatin condensation in up to 25% of cells (Figures 6 and 7). Frequency of nuclear fragmentation after WGA coculture varied

 © 1999 Cancer Research Campaign

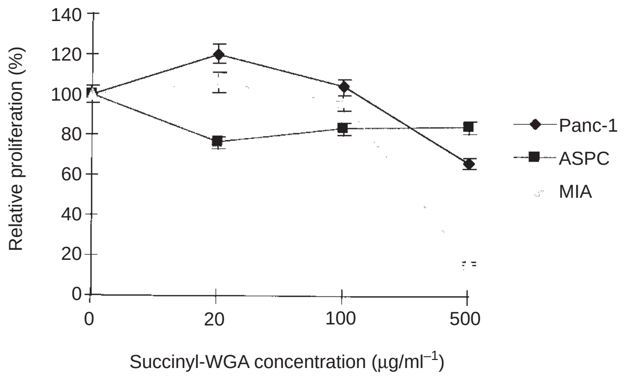

**Figure 3** Succinyl-WGA effect. Cell proliferation of 3 pancreatic cancer cell lines (Panc-1, ASPC, MIA) is shown after 48-hour exposure to succinyl-WGA at various concentrations. Bars depict standard deviation

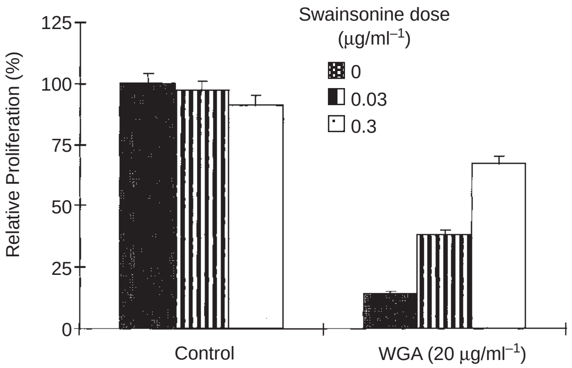

**Figure 4** Swainsonine effect. Proliferation of cell line Panc-1 in the presence of WGA was tested after pretreatment with swainsonine at different concentrations. Bars depict standard deviation

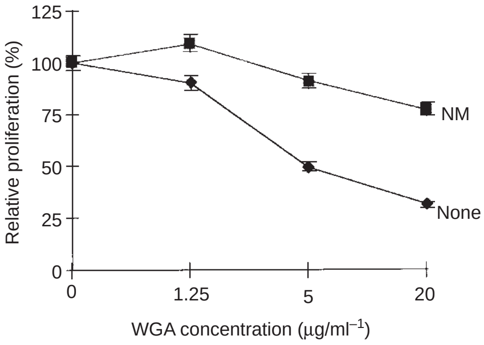

**Figure 5** Neuraminidase pretreatment. Proliferation of cell line Panc-1 in the presence of WGA was tested after pretreatment with neuraminidase (NM), compared to untreated cells (none). Bars depict standard deviation between cell lines. In addition, presence of WGA led to an absence of mitotic nuclei in all cell lines tested. Nuclear fragmentation after ConA exposure was measurable, but less than after WGA treatment. PHA-L and succWGA did not generate similar nuclear effects. A dose-dependent release of BrdU-labelled DNA into the culture supernatant was seen after WGA exposure, but not after ConA or PHA-L exposure (Figure 8).

### FITC-conjugated lectins

Exposure of pancreatic cancer cells to FITC-conjugated WGA at 4°C led to intense cell surface membrane binding by all cells. After various intervals at 37°C this binding pattern was altered. Increased fluorescent activity throughout the cytoplasm was noted within 10–30 min of exposure (Figure 9). Subsequently, preferred intracellular localization at the perinuclear zone and at the cell nucleus could be identified. Maximal nuclear fluorescence was seen 24 h after exposure. FITC-conjugated succWGA, used as control, led to a weaker initial membrane binding, but showed cellular internalization features at 37°C as well (Figure 9). Increased fluorescence, however, did not seem to involve the nucleus, but remained within the cytoplasm even after 48 h.

### Immobilized WGA

Panc-1 and MIA cells grown on plastic tissue culture plates precoated with WGA or ConA did not exhibit a decreased proliferation activity compared to cells grown on control plates (Figure 10). In the case of cell line BxPC, however, a small decrease in proliferative capability was noted. This decreased cell growth was similar in WGA and ConA precoated plates. Under inversion microscopy, BxPC cells on plates precoated with WGA or ConA showed an altered growth pattern with loss of their normal tendency to form multicell aggregates.

A

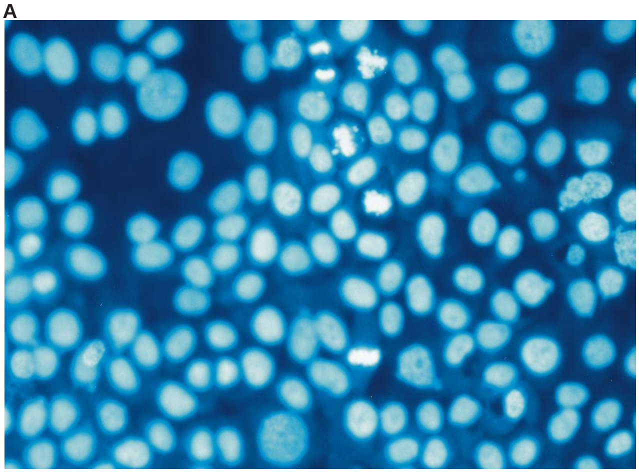

B

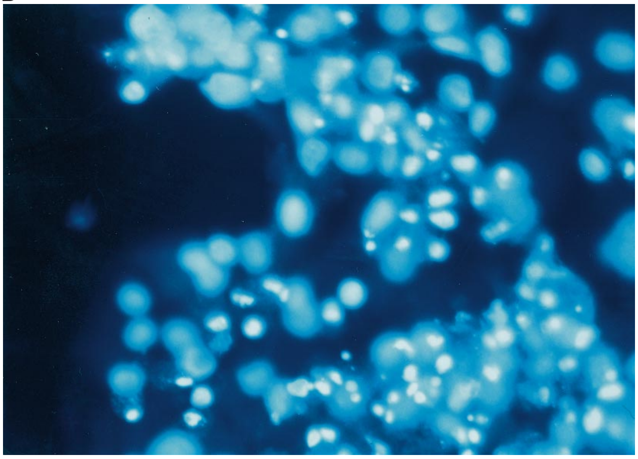

**Figure 6** DAPI stain of cells after WGA exposure. DAPI chromatin stain of cell line BxPC before (**A**) and 48 h after (**B**) exposure to WGA at 20 µg ml$^{-1}$

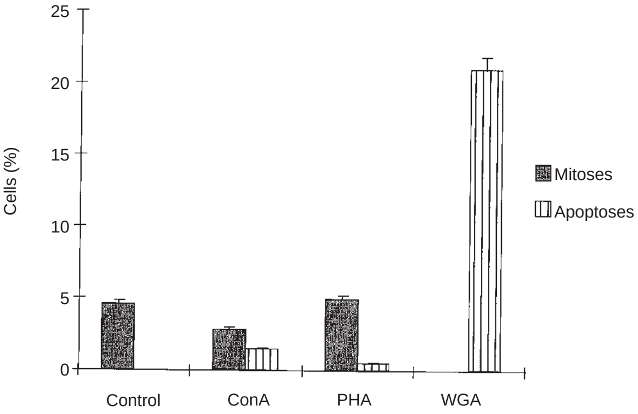

**Figure 7** Apoptotic frequencies. Frequencies of mitotic and apoptotic nuclear formation in BxPC cells after 48 hours of exposure to lectins at 20 µg ml$^{-1}$. Bars depict standard deviation

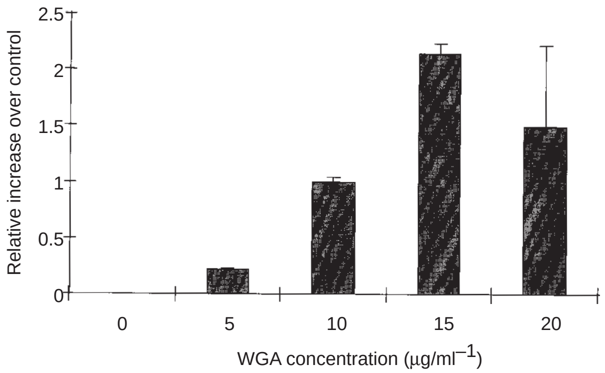

**Figure 8** BrdU analysis. Presence of BrdU-labelled DNA in the supernatant of Panc-1 cells after 48 h of exposure to WGA at various concentrations. Bars depict standard deviation

### Sialic acid binding lectins

Proliferation experiments with cell lines Panc-1 and BxPC were repeated in the presence of sialic acid binding lectins MAA, SNA, and LPA. There was no significant reduction in thymidine incorporation after exposure to these lectins, but a slight increase in cell growth at lower concentrations (Figure 11).

### DISCUSSION

Cancer cells have been shown to express a variety of membrane glycoprotein abnormalities, which in part have been linked to a more aggressive biologic behaviour and metastatic potential (Dennis et al, 1986; Dennis and Laferte, 1987; Yagel et al, 1989; Gorelik et al, 1995). While some of these surface carbohydrate changes in cancer cells reflect specific known genetic alterations such as K-*ras* activation (Wojciechowicz et al, 1995; Schwarz et al, 1996) or loss of the H2-Kb gene (Gorelik et al, 1993), their exact role in carcinogenesis and metastasis remains unclear. Various lectins with specific carbohydrate binding properties have been used to identify specific cell surface carbohydrate compositions in normal or neoplastic cells (Dansey et al, 1988; Langkilde et al, 1989b, 1989c; Aoki et al, 1990; Hohenberger et al, 1990; Kakeji et al, 1994; Schumacher et al, 1994; Takahashi et al, 1994; Mody et al, 1995). WGA has been shown to react with cell membrane glycoproteins in a wide variety of neoplastic tissues (Willmott and Simpson, 1983; Walker, 1984; Kellokumpu et al, 1986; Dansey et al, 1988; Langkilde et al, 1989a; Bresalier et al, 1990; Welch et al, 1990; Mody et al, 1995). When WGA was used to generate lectin-resistant clones of cultured cancer cells, most resulting clones showed a remarkable loss of metastatic capabilities (Stanley et al, 1980; Kerbel et al, 1982; Dennis and Laferte, 1986; Ishikawa et al, 1988; Ishikawa and Kerbel, 1989; Kim et al, 1993). The abnormal truncation of asparagine-linked cell surface carbohydrate with loss of sialylated poly-N-acetyllactosamine complexes and the resulting increase in adherence to laminin, fibronectin and type-4 collagen appears to account for this loss of metastatic potential in WGA-resistant clones (Finne et al, 1980, 1982; Dennis, 1985, 1986a). Therefore, the determination of the presence of sialic acid binding sites on cancer cell surface carbohydrate may carry important functional information.

Sialic acid is a component of many cellular membrane glycoproteins in the normal pancreas (Ronzio et al, 1978; Muresan and Constantinescu, 1985; Willemer et al, 1990; Kameda et al, 1993). It is unclear whether neoplastic progression of pancreatic acinar cells leads to an increase in cell surface sialic acid binding, as determined by WGA reactivity (Skutelsky et al, 1987; Ching et al, 1988). Our results of this study confirm ubiquitous, strong sialic acid reactivity of crude membrane isolates in nine cultured human pancreatic carcinoma cell lines. Furthermore, when these cells are exposed to WGA in vitro, we demonstrate evidence for a dose-dependent WGA-induced toxicity. While WGA has been shown to induce lectin-dependent cell-mediated cytotoxicity against some tumour cells (Kurisu et al, 1980; Ogawara et al, 1985, 1987; Mody et al, 1995), a direct lectin-mediated cancer cell toxicity has not been studied extensively (Gorelik, 1994). Kim et al (1993) have found that toxicity to WGA and *Griffonia simplicifolia* 1-B4 lectins required not only cell surface binding sites, but internalization in order to occur. Both lectins had to be mobile to cause apoptotic cell death in BL6 melanoma clones. These findings are complementary to our results. All pancreatic cancer cells studied presented multiple membrane-bound glycoprotein binding sites for WGA. After initial membrane binding, there was rapid incorporation of WGA into the cells, and WGA immobilized on the plastic dish did not cause similar toxicity. Nuclear condensation and release of fragmented DNA are consistent with an apoptotic process. This strong sensitivity of pancreatic cancer cells to WGA-mediated apoptosis is the more remarkable, as of 15 non-pancreatic cell lines only four exhibited comparable WGA toxicity (results not shown).

The exact mechanisms involved in WGA-induced cancer cell death, however, remain to be clarified. Lack of similar toxicity by succinylated WGA, susceptibility to swainsonine pretreatment, and toxicity inhibition after neuraminidase pretreatment of cells lead us to believe that this effect is initially mediated through WGA binding to sialic acid moieties on cell surface glycoproteins. While the bound lectin subsequently appears to be internalized into the cell, and localizes to the nucleus within as little as 30 min, the resulting biochemical events remain elusive. Interestingly, however, WGA reactivity with components of nuclear membrane has been studied extensively (Kramer and Canellakis, 1979; Jett and Jamieson, 1981; Yoneda et al, 1987; Burrus et al, 1988; Meikrantz et al, 1991; Vannier-Santos et al, 1991; Moore and Blobel, 1992; Sterne-Marr et al, 1992; Kita et al, 1993; Maison

 © 1999 Cancer Research Campaign

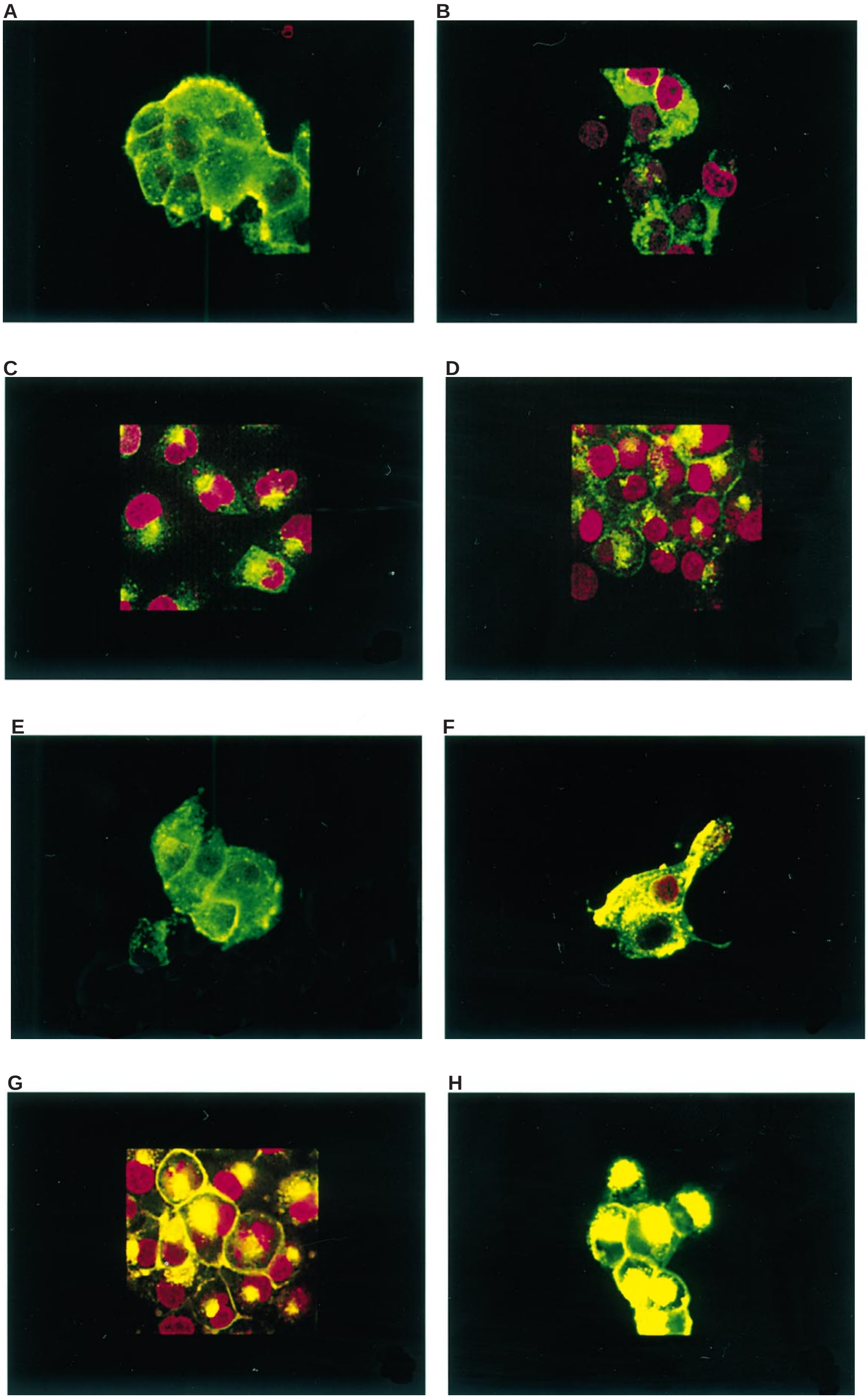

**Figure 9** Fluorescent histolocalization – time course. Confocal microscopy images of Panc-1 cells after exposure to FITC-labelled succinylated WGA (upper panel, **A**–**D**) or WGA (lower panel, **E**–**H**). The four images in each panel (from left to right) were taken at 30 min (**A** and **E**), 6 h (**B** and **F**), 12 h (**C** and **G**) and 24 h of lectin exposure (**D** and **H**). Areas of lectin binding appear green or dark yellow, dependent of the binding intensity. Nuclei were counterstained with propidium iodide (red areas). Accumulation of lectin at the nucleus results in bright yellow fluorescence. While both lectins appear in the cytoplasm as early as 30 min after exposure, only WGA localizes to the perinuclear zone and to the nucleus within the subsequent 24 h.

et al, 1993; Radu et al, 1993; Miller and Hanover, 1994; Beltinger et al, 1995; Dargemont et al, 1995). Accordingly, several glycoprotein components of nuclear pore complexes show WGA binding reactivity, and active transport events at the nuclear pore complex are interrupted after WGA binding (Moore and Blobel, 1992; Sterne-Marr et al, 1992; Kita et al, 1993; Michaud and Goldfarb, 1993; Radu et al, 1993; Adam and Adam, 1994; Bustamante et al, 1994; Miller and Hanover, 1994; Heese-Peck

*© 1999 Cancer Research Campaign* 

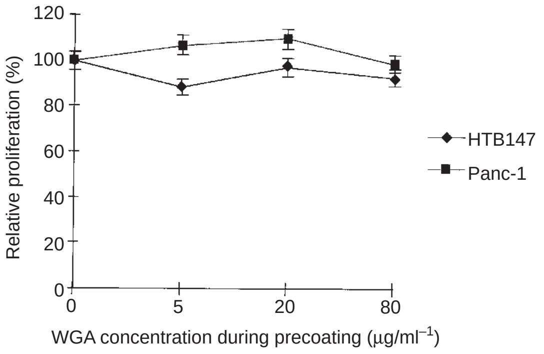

**Figure 10** Immobilized WGA effect. Proliferation of pancreatic cancer cell lines was analysed in untreated plastic tissue culture plates precoated with WGA at various concentrations. Bars depict standard deviation

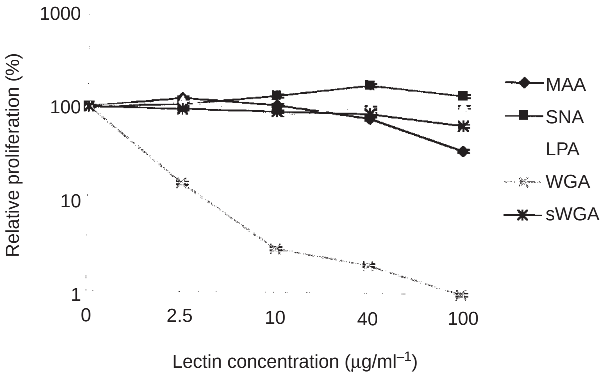

**Figure 11** Sialic acid binding lectins. Proliferation of cell line Panc-1 was tested in the presence of various concentrations of MAA, SNA and LPA. WGA and sWGA were tested as well. Data are presented on an ordinate logarithmic scale. Bars depict standard deviation et al, 1995). We speculate that this nuclear relocation of WGA induces mechanisms operative in the subsequent manifestation of cell death.

The events that fall between surface membrane binding of WGA and the nuclear localization are equally speculative. Evidence from other in-vitro model systems exists, according to which direct WGA-mediated toxicity takes place during mitosis (Lustig et al, 1980), endocytosis of the WGA–receptor complex is directed to the perinuclear region (Kramer and Canellakis, 1979), and intermediate-length filaments such as vimentin exhibit WGA reactivity during prometaphase (Maison et al, 1993). Whether WGA binding to any other cell organelles is involved in the induction of toxicity, is unknown. Binding to cell surface receptor sites is necessary for lectin-induced cytotoxicity, and the receptor number appears to be an important determinant of susceptibility (Schwarz et al, 1996). However, surface binding alone is not sufficient to trigger the cell death response. We assume, that in order to exert a cytotoxic effect, the lectin has to be incorporated into the cell, which is the step that is limited by the number of cell surface structures that carry the ability to facilitate lectin incorporation. Once incorporated, redistribution apparently is a characteristic feature before cell death initiation, and this redistribution may likely involve specific binding steps to other glycoproteins. Although all pancreatic cancer cell lines tested displayed strong membrane WGA binding, the ultimate apoptotic susceptibility was slightly more variable, opening the possibility for additional parameters of ‘internal susceptibility’ to lectin-mediated apoptosis. Accordingly, not all cells exhibiting strong WGA surface binding would be highly susceptible to its toxic effects, while on the other hand WGA membrane binding appears necessary to initiate the apoptotic response. Two indications for this hypothesis exist. Firstly, as lectins that bind only to sialic acid do not mediate similar cell death in pancreatic cancer cell lines, and as succinylated WGA can mediate toxicity in very high concentrations (which may overcome the initially limited GlcNAc-related incorporation step), this intracellular distribution step may depend on GlcNAc binding sites in order to initiate an apoptotic response. WGA binding to nuclear pore complexes have been linked to binding at GlcNAc moieties in other cell models of nuclear transport blockade (Miller and Hanover, 1994; Heese-Peck et al, 1995). Secondly, WGA-resistant murine cells do not always lose their WGA membrane binding affinity after prolonged exposure to the lectin, and may have been selected through a down-regulation or loss of intracellular lectin processing or binding events (Tao et al, 1983; Kim et al, 1993). That both mechanisms are present in all pancreatic cancer cell lines tested is without doubt, as all nine cultured cell lines carry a uniformly strong membrane binding affinity, a subsequent nearly complete nuclear relocalization, and a strong toxic response mediated through apoptosis.

In conclusion, WGA induces toxicity against human pancreatic cancer cells in vitro in a dose-dependent manner. The necessary steps appear to involve WGA binding to sialic acid residues of cell surface glycoproteins, internalization of the lectin, and subsequent localization to the nucleus that itself does not appear to be solely mediated through sialic acid binding. The cell death mode is consistent with apoptosis. Further insight into this mechanism may aid in understanding the complexity of the apoptotic response to therapeutic interventions in human pancreatic cancer.

### ACKNOWLEDGEMENTS

We thank Drs Murray Brennan and Lew Freedman for their scientific support and for their review of the manuscript.

### REFERENCES

Adam EJ and Adam SA (1994) Identification of cytosolic factors required for nuclear location sequence-mediated binding to the nuclear envelope. *J Cell Biol* **125**: 547–555

Aoki D, Nozawa S, Iizuka R, Kawakami H and Hirano H (1990) Differences in lectin binding patterns of normal endometrium and endometrial adenocarcinoma, with special reference to staining with *Ulex europeus* agglutinin 1 and peanut agglutinin. *Gynecol Oncol* **37**: 338–345

Beltinger C, Saragovi HU, Smith RM, Le Sauteur L, Shah N, De Dionisio L, Christensen L, Raible A, Jarett L and Gewirtz AM (1995) Binding, uptake, and intracellular trafficking of phosphorothioate-modified oligodeoxynucleotides. *J Clin Invest* **95**: 1814–1823

Bresalier RS, Rockwell RW, Dahiya R, Duh QY and Kim YS (1990) Cell surface sialoprotein alterations in metastatic murine colon cancer cell lines selected in an animal model for colon cancer metastasis. *Cancer Res* **50**: 1299–1307

Burrus GR, Schmidt WN, Briggs JA, Hnilica LS and Briggs RC (1988) Lectin-binding proteins in nuclear preparations from rat liver and malignant tumors. *Cancer Res* **48**: 551–555

Bustamante JO, Liepins A and Hanover JA (1994) Nuclear pore complex ion channels (review). *Mol Membr Biol* **11**: 141–150

Ching CK, Black R, Helliwell T, Savage A, Barr H and Rhodes JM (1988) Use of lectin histochemistry in pancreatic cancer. *J Clin Pathol* **41**: 324–328

© 1999 Cancer Research Campaign

Dansey R, Murray J, Ninin D and Bezwoda WR (1988) Lectin binding in human breast cancer: clinical and pathologic correlations with fluorescein-conjugated peanut, wheat germ and concanavalin A binding. *Oncology* **45**: 300–302

Dargemont C, Schmidt-Zachmann MS and Kuhn LC (1995) Direct interaction of nucleoporin p62 with mRNA during its export from the nucleus. *J Cell Sci* **108**: 257–263

Dennis JW (1985) Partial reversion of the metastatic phenotype in a wheat germ agglutinin-resistant mutant of the murine tumor cell line MDAY-D2 selected with *Bandeiraea simplicifolia* seed lectin. *J Natl Cancer Inst* **74**: 1111–1120

Dennis JW (1986*a*) Different metastatic phenotypes in two genetic classes of wheat germ agglutinin-resistant tumor cell mutants. *Cancer Res* **46**: 4594–4600

Dennis JW (1986*b*) Effects of swainsonine and polyinosinic:polycytidylic acid on murine tumor cell growth and metastasis. *Cancer Res* **46**: 5131–5136

Dennis JW and Laferte S (1986) Co-reversion of a lectin-resistant mutation and non-metastatic phenotype in murine tumor cells. *Int J Cancer* **38**: 445–450

Dennis JW and Laferte S (1987) Tumor cell surface carbohydrate and the metastatic phenotype. *Cancer Metastasis Rev* **5**: 185–204

Dennis JW and Laferte S, Fukuda M, Dell A and Carver JP (1986) Asn-linked oligosaccharides in lectin-resistant tumor-cell mutants with varying metastatic potential. *Eur J Biochem* **161**: 359–373

Dennis JW, Laferte S, Waghorne C, Breitman ML and Kerbel RS (1987) Beta 1–6 branching of Asn-linked oligosaccharides is directly associated with metastasis. *Science* **236**: 582–585

Dennis JW, Koch K and Beckner D (1989) Inhibition of human HT29 colon carcinoma growth in vitro and in vivo by swainsonine and human interferon-alpha 2. *J Natl Cancer Inst* **81**: 1028–1033

Finne J, Tao TW and Burger MM (1980) Carbohydrate changes in glycoproteins of a poorly metastasizing wheat germ agglutinin-resistant melanoma clone. *Cancer Res* **40**: 2580–2587

Finne J, Burger MM and Prieels JP (1982) Enzymatic basis for a lectin-resistant phenotype: increase in a fucosyltransferase in mouse melanoma cells. *J Cell Biol* **92**: 277–282

Gorelik E (1994) Mechanism of cytotoxic activity of lectins. *Trends Glycosc Glycotechnol* **6**: 435–445

Gorelik E, Kim M, Duty L, Henion T and Galili U (1993) Control of metastatic properties of BL6 melanoma cells by H-2Kb gene: immunological and nonimmunological mechanisms. *Clin Exp Metastasis* **11**: 439–452

Gorelik E, Duty L, Anaraki F and Galili U (1995) Alterations of cell surface carbohydrates and inhibition of metastatic property of murine melanomas by alpha 1,3 galactosyltransferase gene transfection. *Cancer Res* **55**: 4168–4173

Heese-Peck A, Cole RN, Borkhsenious ON, Hart GW and Raikhel NV (1995) Plant nuclear pore complex proteins are modified by novel oligosaccharides with terminal N-acetylglucosamine. *Plant Cell* **7**: 1459–1471

Ho JJ, Chung YS, Fujimoto Y, Bi N, Ryan W, Yuan SZ, Byrd JC and Kim YS (1988) Mucin-like antigens in a human pancreatic cancer cell line identified by murine monoclonal antibodies SPan-1 and YPan-1. *Cancer Res* **48**: 3924–3931

Hohenberger P, Liewald F, Schlag P and Herfarth C (1990) Lectins and immunohistochemistry of colorectal cancer, its recurrences and metastases. *Eur J Surg Oncol* **16**: 289–297

Ishikawa M and Kerbel RS (1989) Characterization of a metastasis-deficient lectin-resistant human melanoma mutant. *Int J Cancer* **43**: 134–139

Ishikawa M, Dennis JW, Man S and Kerbel RS (1988) Isolation and characterization of spontaneous wheat germ agglutinin-resistant human melanoma mutants displaying remarkably different metastatic profiles in nude mice. *Cancer Res* **48**: 665–670

Jett M and Jamieson GA (1981) Comparison of binding sites for wheat germ agglutinin on Raji lymphoblastoid cells and their isolated nuclei and plasma membranes. *Biochemistry* **20**: 5221–5226

Kakeji Y, Maehara Y, Tsujitani S, Baba H, Ohno S, Watanabe A and Sugimachi K (1994) *Helix pomatia* agglutinin binding activity and lymph node metastasis in patients with gastric cancer. *Semin Surg Oncol* **10**: 130–134

Kameda Y, Hirota C and Miyauchi R (1993) Staining of pancreatic centroacinar cells, liver bile canaliculi and testicular Leydig cells with a monoclonal antibody against adrenocortical cells. *Cell Tissue Res* **272**: 407–416

Kellokumpu I, Karhi K and Andersson LC (1986) Lectin-binding sites in normal, hyperplastic, adenomatous and carcinomatous human colorectal mucosa. *Acta Pathol Microbiol Immunol Scand [a]* **94**: 271–280

Kerbel RS, Dennis JW, Largarde AE and Frost P (1982) Tumor progression in metastasis: an experimental approach using lectin resistant tumor variants. *Cancer Metastasis Rev* **1**: 99–140

Kim M, Rao MV, Tweardy DJ, Prakash M, Galili U and Gorelik E (1993) Lectin-induced apoptosis of tumour cells. *Glycobiology* **3**: 447–453

Kita K, Omata S and Horigome T (1993) Purification and characterization of a nuclear pore glycoprotein complex containing p62. *J Biochem* **113**: 377–382

Kramer RH and Canellakis ES (1979) The surface glycoproteins of the HeLa cell. Internalization of wheat germ agglutinin-receptors. *Biochim Biophys Acta* **551**: 328–348

Kurisu M, Yamazaki M and Mizuno D (1980) Induction of macrophage-mediated tumor lysis by the lectin wheat germ agglutinin. *Cancer Res* **40**: 3798–3803

Laemmli UK (1970) Cleavage of structural proteins during the assembly of the head of bacteriophage T4. *Nature* **227**: 680–685

Langkilde NC, Hastrup J, Olsen S, Wolf H and Orntoft TF (1989*a*). Immunohistochemistry and cytochemistry of experimental rat bladder cancer: binding of the lectins PNA and WGA and of a Le(Y) mouse monoclonal antibody. *J Urol* **141**: 981–986

Langkilde NC, Wolf H and Orntoft TF (1989*b*) Binding of wheat and peanut lectins to human transitional cell carcinomas. Correlation with histopathologic grade, invasion, and DNA ploidy. *Cancer* **64**: 849–853

Langkilde NC, Wolf H and Orntoft TF (1989*c*) Lectinohistochemistry of human bladder cancer: loss of lectin binding structures in invasive carcinomas. *Apmis* **97**: 367–373

Lustig S, Fishman P, Djaldetti M and Pluznik DH (1980) Topographic changes of membrane receptors for wheat germ agglutinin during the cell cycle and their relation to cytolysis. *Exp Cell Res* **129**: 321–328

McClay DR, Wessel GM and Marchase RB (1981) Intercellular recognition: quantitation of initial binding events. *Proc Natl Acad Sci USA* **78**: 4975–4979

Maison C, Horstmann H and Georgatos SD (1993) Regulated docking of nuclear membrane vesicles to vimentin filaments during mitosis. *J Cell Biol* **123**: 1491–1505

Maylie-Pfenninger MF and Jamieson JD (1979) Distribution of cell surface saccharides on pancreatic cells. II. Lectin-labeling patterns on mature guinea pig and rat pancreatic cells. *J Cell Biol* **80**: 77–95

Meikrantz W, Smith DM, Sladicka MM and Schlegel RA (1991) Nuclear localization of an O-glycosylated protein phosphotyrosine phosphatase from human cells [published erratum appears in *J Cell Sci* 1991 May; 99 (Pt 1): preceding 1]. *J Cell Sci* **98**: 303–307

Michaud N and Goldfarb DS (1993) Most nuclear proteins are imported by a single pathway. *Exp Cell Res* **208**: 128–136

Miller MW and Hanover JA (1994) Functional nuclear pores reconstituted with beta 1-4 galactose-modified O-linked N-acetylglucosamine glycoproteins. *J Biol Chem* **269**: 9289–9297

Mody R, Joshi S and Chaney W (1995) Use of lectins as diagnostic and therapeutic tools for cancer. *J Pharmacol Toxicol Methods* **33**: 1–10

Monsigny M, Sene C, Obrenovitch A, Roche AC, Delmotte F and Boschetti E (1979) Properties of succinylated wheat-germ agglutinin. *Eur J Biochem* **98**: 39–45

Monsigny M, Roche AC, Sene C, Maget-Dana R and Delmotte F (1980) Sugar-lectin interactions: how does wheat-germ agglutinin bind sialoglycoconjugates? *Eur J Biochem* **104**: 147–153

Moore MS and Blobel G (1992) The two steps of nuclear import, targeting to the nuclear envelope and translocation through the nuclear pore, require different cytosolic factors. *Cell* **69**: 939–950

Muresan V and Constantinescu MC (1985) Distribution of sialoglycoconjugates on the luminal surface of the endothelial cell in the fenestrated capillaries of the pancreas. *J Histochem Cytochem* **33**: 474–476

Ogawara M, Sone S and Ogura T (1987) Human alveolar macrophages: wheat germ agglutinin-dependent tumor cell killing. *Jpn J Cancer Res* **78**: 288–295

Ogawara M, Utsugi T, Yamazaki M and Sone S (1985) Induction of human monocyte-mediated tumor cell killing by a plant lectin, wheat germ agglutinin. *Jpn J Cancer Res* **76**: 1107–1114

Radu A, Blobel G and Wozniak RW (1993) Nup155 is a novel nuclear pore complex protein that contains neither repetitive sequence motifs nor reacts with WGA. *J Cell Biol* **121**: 1–9

Ronzio RA, Kronquist KE, Lewis DS, MacDonald RJ, Mohrlok SH and O'Donnell J, Jr (1978) Glycoprotein synthesis in the adult rat pancreas. IV. Subcellular distribution of membrane glycoproteins. *Biochim Biophys Acta* **508**: 65–84

Schumacher U, Adam E, Flavell DJ, Boehm D, Brooks SA and Leathem AJ (1994) Glycosylation patterns of the human colon cancer cell line HT-29 detected by *Helix pomatia* agglutinin and other lectins in culture, in primary tumours and in metastases in SCID mice. *Clin Exp Metastasis* **12**: 398–404

Schwarz RE, Wojciechowicz DC, Park PY and Paty PB (1996) Phytohemagglutinin-L (PHA-L) lectin surface binding of N-linked beta 1-6 carbohydrate and its relationship to activated mutant ras in human pancreatic cancer cell lines. *Cancer Lett* **107**: 285–291

Skutelsky E, Alroy J, Ucci AA, Carpenter JL and Moore FM (1987) Modulation of carbohydrate residues in regenerative nodules and neoplasms of canine and feline pancreas. *Am J Pathol* **126**: 25–32

© 1999 Cancer Research Campaign

Sowa M, Kato Y, Yamamoto S, Satake K, Kamino K and Umeyama K (1987) A comparative study of experimental and human pancreatic carcinoma with special reference to histochemical findings. *Jpn J Surg* **17**: 493–506

Stanley P, Sudo T and Carver JP (1980) Differential involvement of cell surface sialic acid residues in wheat germ agglutinin binding to parental and wheat germ agglutinin-resistant Chinese hamster ovary cells. *J Cell Biol* **85**: 60–69

Sterne-Marr R, Blevitt JM and Gerace L (1992) O-linked glycoproteins of the nuclear pore complex interact with a cytosolic factor required for nuclear protein import. *J Cell Biol* **116**: 271–280

Takahashi H, Oyaizu T, Fujita Y and Tsubura A (1994) Lectin-binding profiles in MNNG-induced shrew esophageal carcinomas. *Anticancer Res* **14**: 1569–1572

Tao TW, Calderwood S and Hahn GM (1983) Stable heat-resistant clones selected from wild-type and surface variants of B-16 melanoma. *Int J Cancer* **32**: 533–535

Towbin H, Staehelin T and Gordon J (1979) Electrophoretic transfer of proteins from polyacrylamide gels to nitrocellulose sheets: procedure and some applications. *Proc Natl Acad Sci USA* **76**: 4350–4354

Vannier-Santos MA, Saraiva EM and de Souza W (1991) Nuclear and cytoplasmic lectin binding sites in promastigotes of Leishmania. *J Histochem Cytochem* **39**: 793–800

Walker RA (1984) The binding of peroxidase-labelled lectins to human breast epithelium. II. The reactivity of breast carcinomas to wheat germ agglutinin. *J Pathol* **144**: 101–108

Welch DR, McClure SA, Aeed PA, Bahner MJ and Adams LD (1990) Tumor progression- and metastasis-associated proteins identified using a model of locally recurrent rat mammary adenocarcinomas. *Clin Exp Metastasis* **8**: 533–551

Willemer S, Kohler H, Naumann R, Kern HF and Adler G (1990) Glycoconjugate pattern of membranes in the acinar cell of the rat pancreas. *Histochemistry* **93**: 319–326

Willmott N and Simpson S (1983) Wheat germ agglutinin binding to cells derived from in vivo grown solid tumors. *Anticancer Res* **3**: 401–406

Wojciechowicz DC, Park PY and Paty PB (1995) Beta 1-6 branching of N-linked carbohydrate is associated with K-*ras* mutation in human colon carcinoma cell lines. *Biochem Biophys Res Commun* **212**: 758–766

Wright CS (1992) Crystal structure of a wheat germ agglutinin/glycophorin-sialoglycopeptide receptor complex. Structural basis for cooperative lectin-cell binding. *J Biol Chem* **267**: 14345–14352

Yagel S, Feinmesser R, Waghorne C, Lala PK, Breitman ML and Dennis JW (1989) Evidence that beta 1-6 branched Asn-linked oligosaccharides on metastatic tumor cells facilitate invasion of basement membranes. *Int J Cancer* **44**: 685–690

Yoneda Y, Imamoto-Sonobe N, Yamaizumi M and Uchida T (1987) Reversible inhibition of protein import into the nucleus by wheat germ agglutinin injected into cultured cells. *Exp Cell Res* **173**: 586–595

 *© 1999 Cancer Research Campaign*
